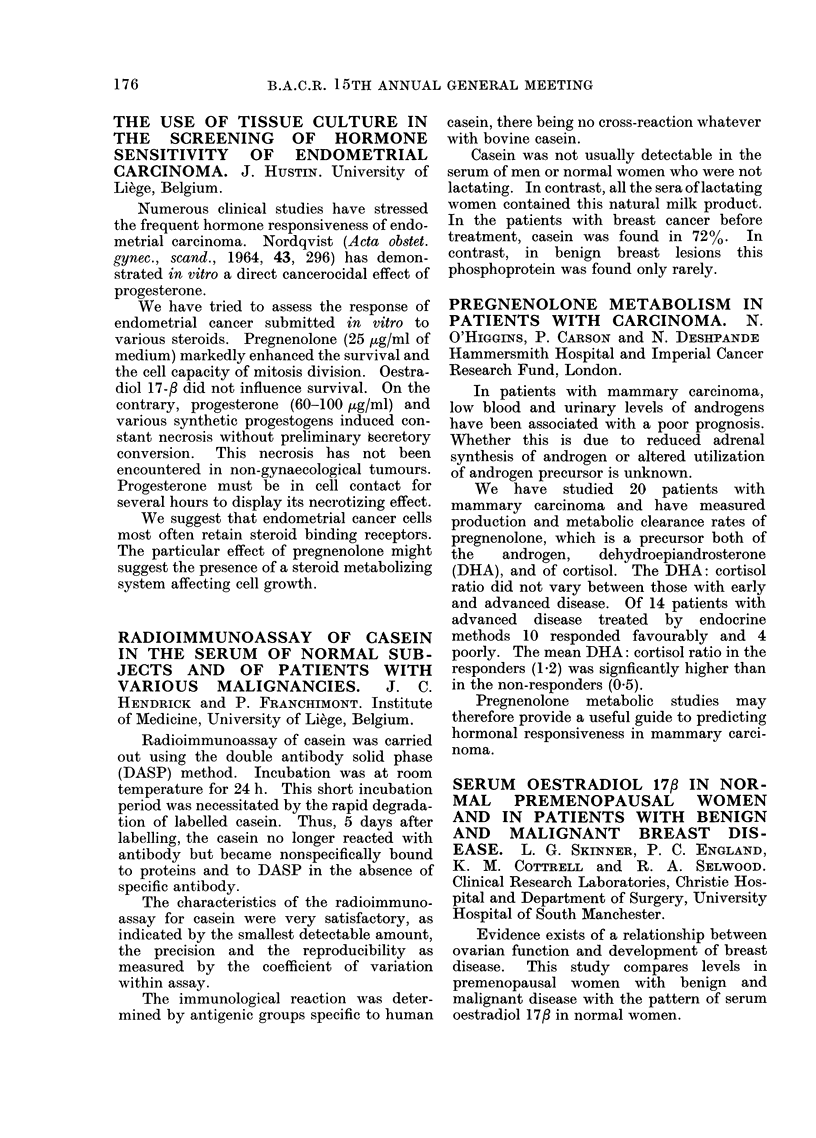# Proceedings: Pregnenolone metabolism in patients with carcinoma.

**DOI:** 10.1038/bjc.1974.143

**Published:** 1974-08

**Authors:** N. O'Higgins, P. Carson, N. Deshpande


**PREGNENOLONE METABOLISM IN PATIENTS WITH CARCINOMA.** N. O'HIGGINS, P. CARSON and N. DESHPANDE Hammersmith Hospital and Imperial Cancer Research Fund, London.

In patients with mammary carcinoma, low blood and urinary levels of androgens have been associated with a poor prognosis. Whether this is due to reduced adrenal synthesis of androgen or altered utilization of androgen precursor is unknown.

We have studied 20 patients with mammary carcinoma and have measured production and metabolic clearance rates of pregnenolone, which is a precursor both of the androgen, dehydroepiandrosterone (DHA), and of cortisol. The DHA: cortisol ratio did not vary between those with early and advanced disease. Of 14 patients with advanced disease treated by endocrine methods 10 responded favourably and 4 poorly. The mean DHA: cortisol ratio in the responders (1·2) was signficantly higher than in the non-responders (0·5).

Pregnenolone metabolic studies may therefore provide a useful guide to predicting hormonal responsiveness in mammary carcinoma.